# Seasonal Variation of the Effects of Phylogenetic Relatedness and Functional Similarity Among Heterospecific Neighbors and Habitat on Seedling Survival in a Subtropical Forest in Gaoligong Mountains, Southwest China

**DOI:** 10.1002/ece3.73021

**Published:** 2026-02-02

**Authors:** Liping Wang, Junjie Wu, Yong Chai, Fengxian Chen

**Affiliations:** ^1^ School of Ecology Hainan University Haikou China; ^2^ College of Agriculture and Biology Liaocheng University Liaocheng China; ^3^ Yunnan Academy of Forestry and Grassland Kunming China; ^4^ College of Agriculture and Biological Science Dali University Dali China

**Keywords:** functional similarity, Gaoligong Mountains, habitat filtering, phylogenetic relatedness, seasonality, seedling survival

## Abstract

Understanding the mechanisms of species coexistence is a long‐standing goal in community ecology. The performance of woody seedlings across different ecological niches has been proposed as a key mechanism in community assembly. Factors influencing seedling survival can be revealed by analyzing neighboring plants in conjunction with their phylogenetic relatedness, functional traits, and the surrounding environment. In the present study, we conducted a seasonal analysis of 936 seedlings for 56 species from a 4‐ha subtropical forest over 3 years in the Gaoligong Mountains, Southwest China. Our aims were to examine the relative effects of neighbor density, habitat conditions, and seasonal climate variability on seedling survival during dry and rainy seasons, and to determine whether the sensitivity of seedling survival responding to neighbor densities and abiotic factors differs between seasons. The findings indicated that the relative importance of neighbor densities, habitat factors as well as seasonal rainfall on seedling survival varied with seasonality. During the rainy season, seedling survival was comparatively less affected by conspecific neighbor density and was mainly negatively influenced by rainfall, whereas habitat factors (including topography, soil properties, and canopy openness) were detrimental for survival to a lesser extent. There is evidence suggesting that phylogenetic negative density dependence (PNDD) and functional negative density dependence (FNDD) are more pronounced in the rainy season. In contrast, the positive effect of canopy openness was more important during the dry season. Our findings further revealed that the effects of PNDD, FNDD, canopy openness, and seasonal rainfall varied widely among species across seasons. Moreover, species differed in their ability to respond to the trade‐offs between canopy openness and rainfall in relation to phylogenetic relatedness and functional dissimilarity during the dry and rainy seasons. Overall, our results demonstrate that seasonality modulates the strength and importance of phylogenetic and functional density dependence, habitat preference, and climate for seedling survival in subtropical forests. The seasonal variations in these effects allow species to maintain coexistence across dry and rainy seasons. Consequently, seasonal variability should be accounted for in future studies in understanding the diversity of forest communities.

## Graphical abstract

Based on seedling dynamic data from a subtropic forest in Southwest China, we found phylogenetic negative density dependence (PNDD) and functional negative density dependence (FNDD) on seedling survival in the rainy season, but not in the dry season. In the dry season, greater canopy openness significantly enhanced seedling survival, whereas in the rainy season, higher rainfall intensity unexpectedly lowered survival. These results collectively indicate a marked seasonal shift in the dominant drivers of seedling dynamics—from strong biotic regulation (PNDD/FNDD) in the rainy season to abiotic facilitation (light availability) in the dry season—thereby demonstrating how temporal niche partitioning may stabilize forest communities.

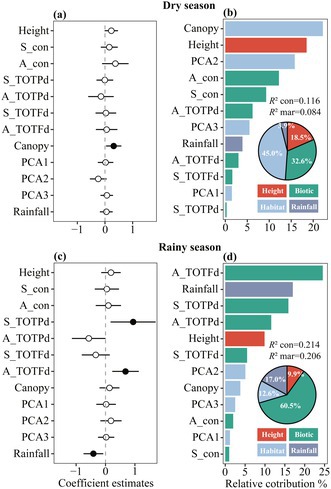

## 1 Introduction

Conspecific negative density dependence (CNDD) is regarded as a central driver in maintaining community assembly and species coexistence (Chen et al. 2019; Wright 2002), wherein individual performance and population growth diminish as the density of conspecific individuals increases (Connell 1971; Janzen 1970). Density‐dependent survival during the seedling stage is increasingly recognized as a crucial filter for forest regeneration. Seedling is a vulnerable life stage and faces greater challenges in the forest understory compared to adult trees (Metz et al. 2023), largely due to their limited physical and chemical defenses, which increase their susceptibility to natural enemies (Descombes et al. 2020; Jia et al. 2018).

Previous studies have primarily focused on taxonomic information, such as species richness and composition (i.e., conspecific or heterospecific), which may ignore phylogenetic and functional dimensions of neighborhood interactions (Swenson et al. 2011). Recent evidence indicates that the strength of neighborhood effects is influenced by both phylogenetic relatedness and functional dissimilarity between focal individuals and heterospecific neighbors (Wang et al. 2020; Wang, Wu, Chai, Sun, et al. 2024). The species' performance declined when they were faced with higher densities of phylogenetically related heterospecific neighbors (phylogenetic negative density dependence, PNDD) (Metz et al. 2010; Zhu et al. 2015). Conversely, the phylogenetic positive density dependence (PPDD) affecting seedling survival was also confirmed in tropical forests (Lebrija‐Trejos et al. 2014; Wu et al. 2016; Zhu et al. 2015). Functional trait similarity among neighboring trees is another crucial factor driving community dynamics. Sharing similar traits and ecological requirements among neighbors can lead to strong competition for resources; then neighbors would appear competitive exclusion and be unlikely to coexist (Darwin 1859; Lebrija‐Trejos et al. 2014; Swenson et al. 2007), generating functional negative density dependence (FNDD). Besides, a positive effect of functional similarity may occur among neighbors (functional positive density dependence, FPDD). This implies that focal species surrounded by neighbors with lower functional divergence (i.e., higher similarity) may enhance survival, likely due to benefit from shared mutualistic networks, ameliorated microclimates, or collective defense (Ames et al. 2020). Thus, assessing the role of phylogenetic relatedness and functional similarity among neighbors on seedling survival is crucial for elucidating the mechanisms underlying neighborhood interaction.

The strength of neighborhood effect and its variation among neighbors are also strongly influenced by surrounding environmental conditions, particularly topography and soil properties. For example, localized elevation gradients lead to spatial environmental heterogeneities, which typically lead to greater soil moisture and fertility in bottomland areas compared to higher elevations (John et al. 2007). Because of terrain gradients in nutrients and water, soils are generally deeper in valleys than on hilltops, with concomitantly higher water availability (Guo et al. 2018). Furthermore, soil acidity and compactness are vital for seedling survival, since they directly affect resource accessibility and soil structure (Soong et al. 2020). Light is another crucial limiting resource that greatly impacts tree growth, survival, and competitive consequences (Song et al. 2020; Yao et al. 2020). This is particularly true for understory seedlings, where light availability is highly heterogeneous in space due to canopy gaps created by treefall. Consequently, local light levels vary substantially with an individual's spatial location within the forest (Chazdon and Fetcher 1984).

Additionally, along with seasonal fluctuations in abiotic conditions, the strength of neighborhood effect on seedling survival may also vary across seasons (Lin et al. 2012; Mensah et al. 2023; Song et al. 2020, 2024). Previous studies have affirmed that the activity of herbivores and pathogens can increase under the wet and warm conditions of the rainy season (Brenes‐Arguedas et al. 2009; Spear et al. 2015), potentially reinforcing the negative effects of natural enemies on seedling survival. However, the key drivers determining seasonal patterns of seedling survival in subtropical forests remain elusive. In forest ecosystems, seasonal climate changes represent a potential driving force for niche partitioning and can greatly influence community dynamics (Yin et al. 2024). Although numerous studies have investigated the effects of daily and interannual precipitation variations on seedling survival (Johnson et al. 2017; Xu et al. 2022), the significance of seasonal precipitation variation as a vital factor influencing seedling dynamics has been less explored.

To date, little is known about whether and how phylogenetic relatedness and functional similarity influence seedling survival across dry and rainy seasons. These species traits may alter in response to seasonal habitat heterogeneity and climate change, and the strength of their effects on seedling survival may also vary among species in seasonal forests (Johnson et al. 2017; Lin et al. 2012; Yao et al. 2020).

Understanding how multiple mechanisms interact to shape seedling survival patterns is essential for elucidating the drivers of community assembly. Here, we evaluate how seedling survival across seasons is influenced by biotic factors (phylogenetic relatedness and functional similarity among neighbors) and abiotic factors (habitat heterogeneity and seasonal climate) in a subtropical forest. We specifically hypothesize that: (i) the relative importance of biotic and abiotic factors on seedling survival varies seasonally at both the community and species levels. (ii) Seedling survival is affected by phylogenetic and functional density dependence, with both the direction (positive or negative) and strength of this dependence varying seasonally. (iii) There is a seasonal difference in the trade‐off between the sensitivity of seedling survival to phylogenetic/functional densities and its sensitivity to abiotic factors.

## 2 Materials and Methods

### 2.1 Study Area and Census Data

**FIGURE 1:**
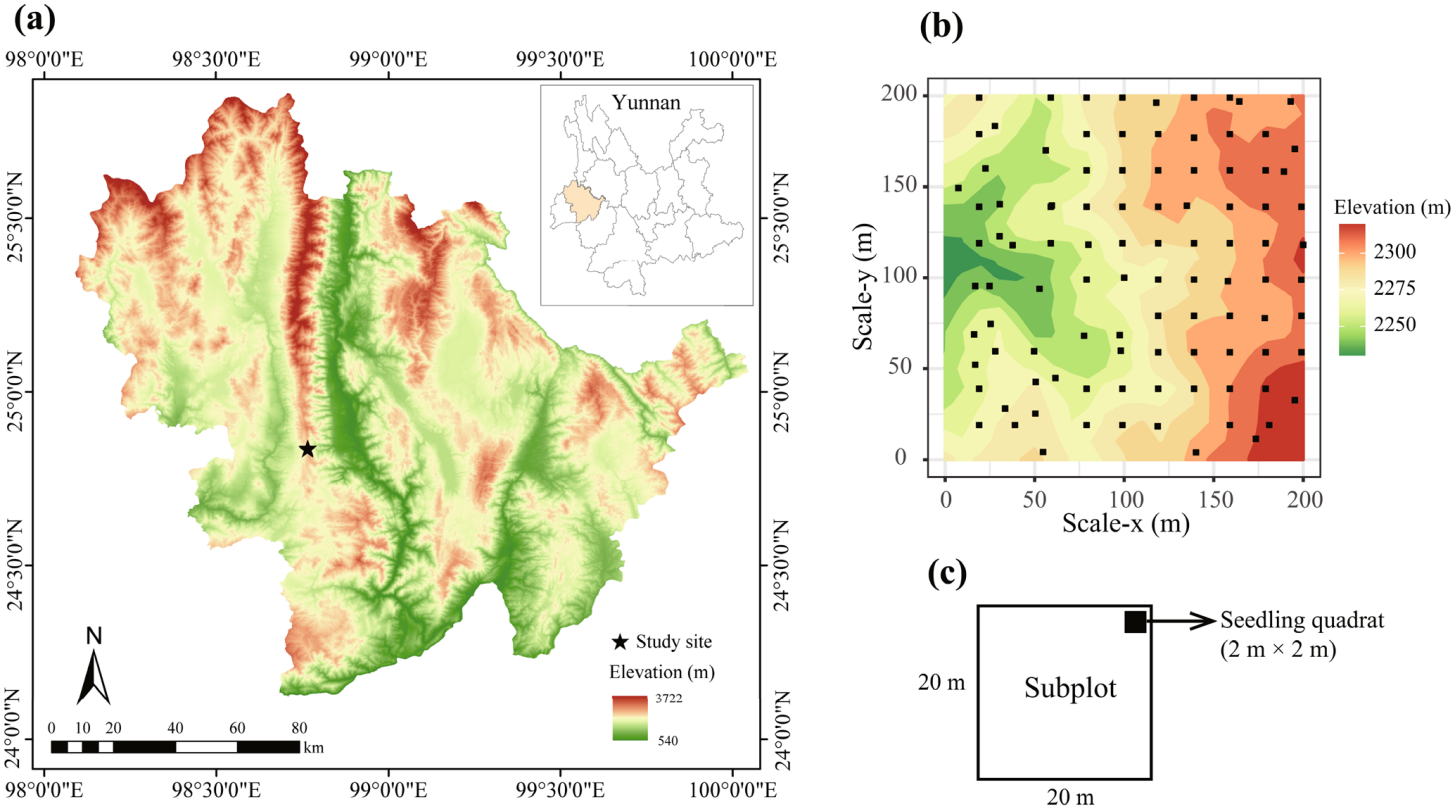
Study site and sampling design. (a) Location of the study area. (b) Topography of the 4‐ha permanent plot and seedling quadrats indicated by black solid squares. (c) Layout of seedling quadrat in subplot.

The study was carried out at a 4‐ha (200 m × 200 m) subtropical forest plot in Gaoligong Mountains, Southwestern China (24°50′9.8″–24°50′17.3″ N, 98°45′53.1″–98°46′1.3″ E; Figure 1a). Established between 2009 and 2010 following the CForBio protocol (Chai et al. 2014), the plot is situated in the core of its vegetation zone and was selected for minimal recent anthropogenic disturbance. Dominant tree species, including *Symplocos ramosissima*, *Eurya pseudocerasifera*, *Polyspora longicarpa*, and *Neolitsea lunglingensis*, are characteristic of and widely reported for the forest (Meng et al. 2013; Wang, Wu, Chai, Li, et al. 2024). The region has a mean annual temperature of 17°C with approximately 1200 mm of precipitation (Chai et al. 2014). The terrain is rugged, being high in the north and low in the south. The soil in the plot is predominantly yellow‐brown loam. The vegetation is a typical mid‐montane humid evergreen broad‐leaved forest. The plot was divided into 100 subplots (20 m × 20 m). All freestanding woody stems with a diameter at breast height (DBH) ≥ 1 cm were tagged, mapped, measured, and identified to species.

Within the plot, we established 100 seedling quadrats in 2020 to monitor seedling performance (Figure 1b). Each quadrat was 4‐m^2^ (2 m × 2 m) and positioned in the top‐right corner of a corresponding 20 m × 20 m subplot (Figure 1c). All seedlings with DBH < 1 cm and height ≥ 20 cm were tagged at the end of each dry (May) and rainy (November) season. During these censuses, we recorded species identity, measured height, and assessed survival status (alive or dead). Seedlings that were monitored from May to October were defined as the survival status in the rainy season, and seedlings monitored from November to April of next year were defined as the survival status in the dry season. In the present study, we analyzed seedling survival data from 2020 to 2022 (five censuses). Seedlings monitored from May to October were assigned to the rainy‐season survival interval, while those monitored from November to April of the following year were assigned to the dry‐season interval. For the present study, we analyzed seedling survival data collected from 2020 to 2022, encompassing five seasonal censuses.

### 2.2 Environmental Variables

To examine the impacts of environmental conditions on seedling survival, we conducted canopy openness, topography, and soil properties. Canopy openness was estimated from hemispherical photographs taken with a Nikon camera (Z5N1933) equipped with an F2.8 12‐mm Senyo round fisheye lens. The photographs were taken 1.3 m above the ground at each seedling quadrat during the winter of 2021. Gap Light Analyzer 2.0 (GLA) software was used to compute canopy openness from these images, which served as a proxy for light availability. We measured topographic variables for each seedling quadrat, including elevation, slope, and convexity. Elevation was recorded at the four corners of a 20 × 20 m subplot, and the mean value was assigned as the quadrat elevation. Slope was defined as the mean angular deviation from the horizontal across the four triangular planes formed by connecting three corners of the quadrat. Convexity was calculated as the elevation of the focal quadrat minus the mean elevation of the eight surrounding quadrats. For edge seedling quadrats, convexity was calculated as the elevation at the center point minus the mean elevation of its four corners.

In May 2022, we collected 300 soil samples from the 100 seedling quadrats. After removing the superficial leaf litter and humus layer at each quadrat, soil was sampled at three random locations (0–5 cm depth) using a split‐core sampler, yielding a composite sample of approximately 500 g per quadrat.

These samples were analyzed for ten soil properties: temperature, moisture, pH, electrical conductivity (EC), organic matter content (OMC), available phosphorus (AP), available potassium (AK), total nitrogen (TN), total phosphorus (TP), and total potassium (TK). Detailed information on the sampling and analyses of soil variables can be found in Wang, Wu, Chai, Sun, et al. (2024).

We used the principal component analysis (PCA) to address the collinearity and reduce dimensionality among soil and topographic variables. The first three principal components explained 66.2% and 66.9% of the variance in the dry and rainy seasons, respectively, and were incorporated into subsequent models. The first principal component (PCA1) was strongly associated with elevation, the second principal component (PCA2) with OMC, and the third principal component (PCA3) with slope in both seasons (Table S1). Canopy openness as an independent factor was not included in the PCA.

### 2.3 Seasonal Rainfall Measurement

The monthly rainfall data from 2020 to 2022 were obtained from the Longling County Meteorological Bureau (24°6′ N, 98°68′ E). For each year, we calculated the total rainfall in each season, where May to October was defined as the rainy season and November to April of the next year was defined as the dry season (Figure S1).

### 2.4 Neighborhood Variables

We defined conspecific seedling density (S_con) as the number of conspecific seedlings within the same 4‐m^2^ (2 m × 2 m) quadrat. Conspecific adult tree density (A_con) was calculated as the sum of the basal area of each stem within a 20 m radius, weighted by the inverse of its distance to the focal seedling quadrat, using the formula:

(1) $$A\_\mathrm{con}=\sum_{i}^{N}\frac{{\mathrm{BA}}_{i}}{{\text{Distance}}_{i}}$$

where *i* identifies stem (DBH ≥ 1 cm), *N* is the adult individual. BA_*i*_ is the basal area (m^2^) of *i*th tree neighbor. Distance_*i*_ is the distance between the focal seedling quadrat and the *i*th tree. We selected the 20‐m radius based on preliminary analyses that compared the Akaike's information criterion (AIC) values of survival models using radii of 5, 10, 15, and 20 m; the 20‐m radius had the lowest AIC value (Table S2).

### 2.5 Phylogeny and Traits

#### 2.5.1 Functional Trait Measurements

Between November 2021 and May 2022, we measured nine leaf functional traits for each species in the plot. The traits included leaf chlorophyll content (Chl), leaf thickness (LT), leaf dry‐matter content (LDMC), leaf area (LA), specific leaf area (SLA), leaf carbon content (C), leaf nitrogen content (N), leaf phosphorus content (P), and leaf potassium content (K). For each species and trait, values were derived from a pooled sample of 25 individuals (5 plants × 5 leaves).

All leaf functional trait measurements followed the standard protocols of Pérez‐Harguindeguy et al. (2013). In the field, leaf samples were wiped clean with paper, placed in plastic bags containing silica gel desiccant, and transported to the laboratory. Leaf fresh weight was determined using an electronic balance. LT was measured at three random points on each of five leaves per sample using a digital micrometer (Electronic Digital Micrometer Comecta, Barcelona, Spain). Chl was estimated using a handheld SPAD meter (Konica Minolta SPAD‐502), and the mean value was taken.

Leaves were then scanned using a digital scanner (Epson Perfection V39), and LA (cm^2^) was determined using leaf 1000 software. For two species with exceptionally large leaves (*Actinodaphne obovata* and *Trevesia palmata*), multiple overlapping scans were performed and digitally merged to obtain the total area. Subsequently, leaves were oven‐dried at 70°C for 72 h to constant mass, and dry mass was weighed. SLA (cm^2^ g^−1^) was calculated as LA divided by leaf dry mass. LDMC (g g^−1^) was calculated as dry mass divided by fresh mass.

For elemental analysis, dried leaf samples were ground to a fine powder. Leaf C was determined by the potassium dichromate volumetric method. Leaf N was measured using an automatic Kjeldahl nitrogen analyzer following the standard NY/T 2419‐2013. Leaf P was assayed by molybdenum‐antimony anti‐spectrophotometry following the standard NY/T 2421‐2013. Leaf K concentration was determined by flame photometry following the standard NY/T 2420‐2013.

#### 2.5.2 Construction of Phylogenetic Tree and Functional Trait Dendrogram

To obtain phylogenetic and functional distances between focal seedlings and neighbors, we first constructed a phylogenetic tree for all study species using the “V.PhyloMaker 2” package (Jin and Qian 2022). Pairwise phylogenetic distances were then derived from this tree using the cophenetic function. For functional traits, each trait was standardized, and a functional trait dendrogram was generated based on trait similarity. From this dendrogram, we calculated a pairwise Euclidean distance matrix to represent functional distances.

To quantify the effects of heterospecific neighbors on focal seedlings, we calculated four neighborhood metrics: the total phylogenetic distance of seedling (S_TOTPd) and adult (A_TOTPd) neighbors, and total functional distance of seedling (S_TOTFd) and adult (A_TOTFd) neighbors. These metrics represent the overall phylogenetic relatedness and functional similarity of the neighborhood surrounding each seedling (Webb et al. 2006). They were derived from the phylogenetic tree and functional dendrogram, respectively, using the “picante” package (Kembel et al. 2010) with the following formula:

(2) $$S\_\text{TOTPd or}\;A\_\text{TOTPd}=\sum_{1}^{i}{\text{phylogenetic}}_{i}\times {\text{abundance}}_{i}$$

(3) $$S\_\text{TOTFd or}\;A\_\text{TOTFd}=\sum_{1}^{i}{\text{functional}}_{i}\times {\text{abundance}}_{i}$$

where ${\text{phylogenetic}}_{i}$ and ${\text{functional}}_{i}$ denote the phylogenetic and functional distances between focal seedling and heterospecific neighbor *i*, separately; abundance_*i*_ is the abundance of that heterospecific neighbor. We used adult neighborhoods with a distance ≤ 20 m for all analyses.

### 2.6 Statistical Analysis

To assess how neighbor densities, habitat variables, and seasonal rainfall differentially affect seedling survival between dry and rainy seasons, and to evaluate the roles of phylogenetic relatedness and functional similarity, we fitted generalized linear mixed models (GLMMs) at the community level using the “lme4” package. The response variable was the survival status of each seedling at the end of a census period (alive = 1, dead = 0). The full set of predictors included selected neighbor‐density variables, habitat factors (PCA‐derived components and canopy openness), and seasonal rainfall. Seedling initial height, a known vital determinant of survival, was log‐transformed and included as a covariate in all models. All continuous variables were standardized before running the model (Gelman and Hill 2006). To account for variation attributable to species identity, spatial location (quadrat), and census year, we included these random effects in the model as follows:

$$y_{\mathrm{ijk}}\sim \text{binomial}\;\left(1,p_{\mathrm{ijk}}\right)$$

(4) $$\begin{array}{cc} \log\left(\frac{p_{\mathrm{ijk}}}{1-p_{\mathrm{ijk}}}\right)\;=\; & \beta_{0}+\beta_{1}{\text{Height}}_{\mathrm{ijk}}+\beta_{2}{\text{Density}}_{\mathrm{ijk}}+\beta_{3}{\text{Phylogeny}}_{\mathrm{ijk}}\\ & +\beta_{4}{\text{Function}}_{\mathrm{ijk}}+\beta_{5}{\text{Habitat}}_{\mathrm{jk}}+\beta_{6}{\text{Rainfall}}_{\mathrm{jk}}\\ & +\gamma_{i}+\varphi_{j}+\mu_{k}+\varepsilon_{\mathrm{ijk}} \end{array}$$

where $y_{\mathrm{ijk}}$ represents the binary survival status (alive or dead) of seedling *i* in quadrat *j* during census year *k*, with $p_{\mathrm{ijk}}$ being the corresponding predicted survival probability. $\beta_{0}$ is the intercept. $\beta_{1}$ to $\beta_{6}$ are the fixed‐effect coefficients for initial seedling height, neighborhood density, phylogenetic distance, functional distance, habitat variables, and seasonal rainfall, respectively. $\gamma_{i}$, $\varphi_{j}$, and $\mu_{k}$ are the random intercepts for species identify, seedling quadrat, and census year, respectively. $\varepsilon_{\mathrm{ijk}}$ is the residual error term.

To assess whether the effects of neighbor densities, habitat variables, and climate on seedling survival differ between dry and rainy seasons at the community level, we first examined the estimated coefficients from the fitted GLMMs. A positive coefficient indicates a favorable effect on seedling survival, whereas a negative coefficient indicates an unfavorable effect. In the context of phylogenetic (S_TOTPd, A_TOTPd) and functional (S_TOTFd, A_TOTFd) distance metrics, a positive coefficient signifies greater distance (i.e., weaker relatedness or similarity) among neighbors, which promotes survival and thus reflects phylogenetic or functional negative density dependence (PNDD or FNDD), and vice versa. We conducted diagnostic tests (including overdispersion statistics and residual checks) to confirm that the GLMM assumptions were satisfied. Additionally, the “glmm.hp” package (Lai et al. 2022, 2023) was used to partition and estimate the relative importance of fixed effects in the GLMMs.

To quantify whether species differed in their responses to neighborhood densities and abiotic factors, we fitted species‐specific random slopes for the continuous variables that were significant in the community‐level models (Chen et al. 2010). Following Bolker et al. (2009), we assessed the significance of adding species‐specific random slopes using likelihood ratio tests. The slope terms and random intercepts were then used to quantify interspecific variation in species‐specific responses to phylogenetic and functional distance, canopy openness, and seasonal rainfall. We applied a false discovery rate (FDR) correction to adjust *P*‐values for multiple comparisons. Paired *t*‐tests were used to evaluate whether the species‐specific coefficients for phylogenetic and functional distances of heterospecific neighbors, canopy openness, and rainfall varied significantly between dry and rainy seasons. Finally, we used separate linear regressions for the dry and rainy seasons to evaluate whether the sensitivity of survival to phylogenetic and functional densities was correlated with its sensitivity to abiotic factors (canopy openness and seasonal rainfall). The sensitivity of survival to varying neighbor densities and abiotic variables represents species along a trade‐off related to survival in the living environment.

All analyses were performed using R version 4.5.1 (R Core Team 2025).

## 3 Results

### 3.1 Seedling Seasonal Dynamics in the 4‐Ha Plot

A total of 936 seedlings belonging to 56 species were monitored across the study period from November 2020 to 2022. Seedling survival was highest during the first rainy season (97.6%), which also had the second‐highest seedling abundance. Survival declined in the subsequent intervals: the first dry season, second dry season, and second rainy season (Table S3, Figure S2). Survival rates varied widely among species (33%–100%) and showed a general decline over time. Across all censuses, *Psychotria morindoides* exhibited the highest seedling recruitment. Throughout the study, recruitment rates consistently remained lower than seedling mortality rates, causing a steady decline in total seedling number over time.

### 3.2 Relative Importance of Biotic and Abiotic Factors Driving Seedling Survival Across Seasons

**FIGURE 2:**
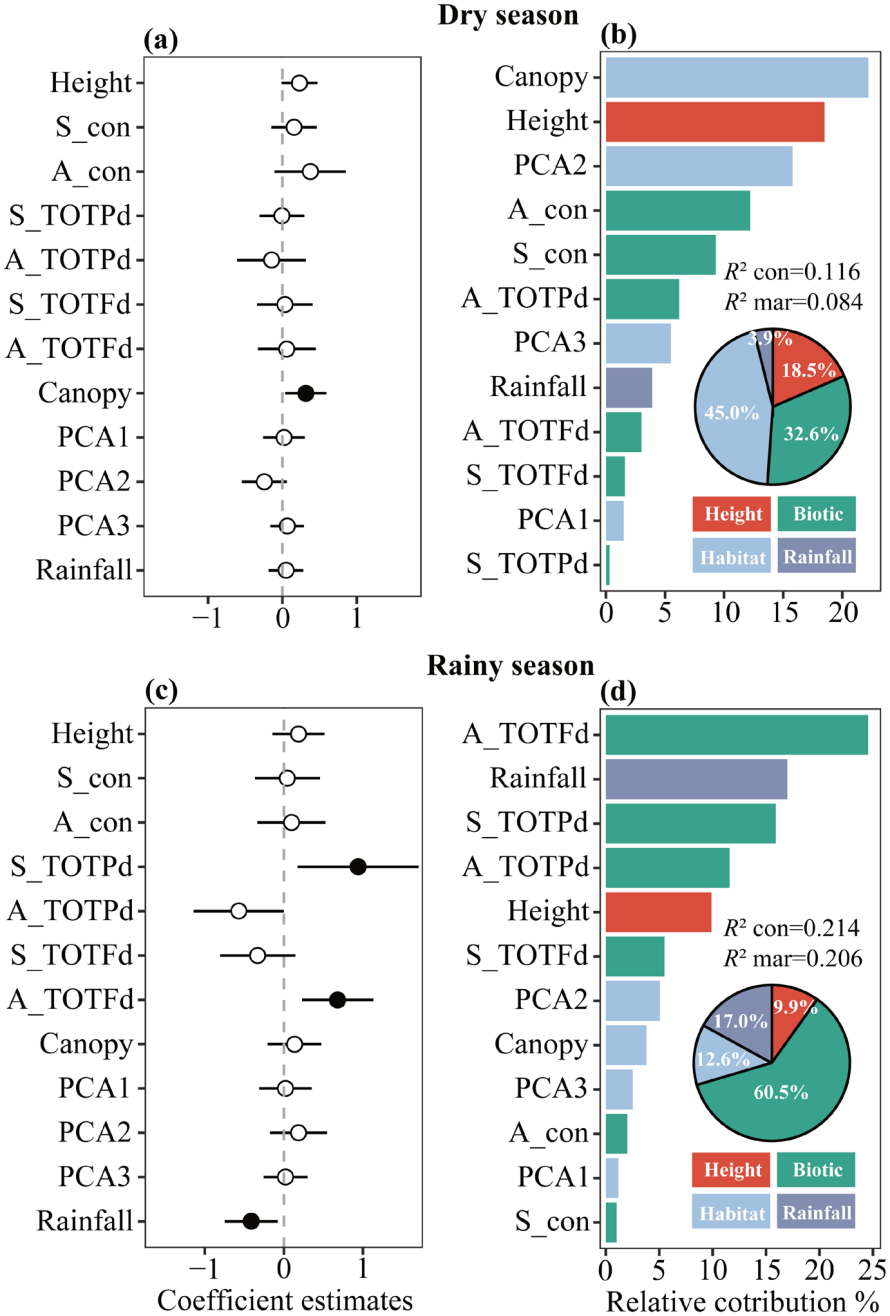
Estimated coefficients (mean ±2 SE) of neighborhood variables, habitat conditions, and climate at the community level on seedling survival in the dry and rainy seasons. (a, c) Filled circles denote significant effects (*p* < 0.05), open circles denote no significance (*p* > 0.05); error bars represent 95% confidence intervals. (b, d) Bar graphs show the percentage contribution of each fixed effect to the model; pie charts depict the relative contribution of predictor groups (seedling height, biotic interactions, habitat factors, seasonal rainfall). Height, initial seedling height; S_con, conspecific seedling density; A_con, conspecific adult tree density; S_TOTPd, total phylogenetic distance of seedling neighbors; A_TOTPd, total phylogenetic distance of adult neighbors; S_TOTFd, total functional distance of seedling neighbors; A_TOTFd, total functional distance of adult neighbors; Canopy, canopy openness; PCA1–PCA3, first three principal components of soil and topographic variables; Rainfall, seasonal rainfall. Conditional *R* ^2^ (*R* ^2^ con) accounts for variance explained by both fixed and random effects; marginal *R* ^2^ (*R* ^2^ mar) accounts for variance explained by fixed effects alone. Sample size: *N* = 936 seedlings.

The relative importance of different drivers of seedling survival varied between dry and rainy seasons (Figure 2, Table S4). Diagnostic checks confirmed the robustness of the fitted models, revealing no significant overdispersion (dry: dispersion = 0.995, *p* = 0.968; rainy: dispersion = 0.996, *p* = 0.984; Figure S3). Seedling height, conspecific seedling and adult neighbor densities, and habitat variables (topographic and soil) had no significant effect on seedling survival during both seasons (all *p* > 0.05; Figure 2a,c). In the dry season, only canopy openness had a significant positive effect on seedling survival (*β* = 0.315, *p* = 0.026). In the rainy season, seedling survival was positively affected by the phylogenetic distance of seedling neighbors (S_TOTPd; *β* = 0.939, *p* = 0.016) and the functional distance of adult neighbors (A_TOTFd; *β* = 0.681, *p* = 0.003), but was negatively influenced by seasonal rainfall (*β* = −0.412, *p* = 0.016). These results indicate that seedlings survived better when surrounded by phylogenetically and functionally distant neighbors and when rainfall was lower in the rainy season. This pattern reflects the effects of PNDD and FNDD on seedling survival during the rainy season.

The total variance in seedling survival explained by the models differed between dry (*R* ^2^ = 0.116) and rainy (*R* ^2^ = 0.214) seasons. In the dry season (Figure 2b), canopy openness was the dominant predictor, accounting for 45.0% of the relative contribution to seedling survival, followed by neighborhood densities (32.6%), initial seedling height (18.5%), and seasonal rainfall (3.9%). In the rainy season (Figure 2d), biotic factors collectively contributed 60.5% of the explained variance, with A_TOTFd (24.6%), S_TOTPd (15.9%), and A_TOTPd (11.6%) being the most influential individual predictors. In contrast, the contribution of initial seedling height was only 9.9%. Among abiotic factors, seasonal rainfall explained 17.0% and habitat factors 12.6%.

In summary, the drivers of seedling survival were fundamentally seasonal: canopy openness prevailed in the dry season, whereas a combination of biotic neighborhood effects (PNDD and FNDD) and lower rainfall dictated survival in the rainy season.

### 3.3 Species‐Level Variation in Responses to Biotic and Abiotic Factors Across Seasons

**FIGURE 3:**
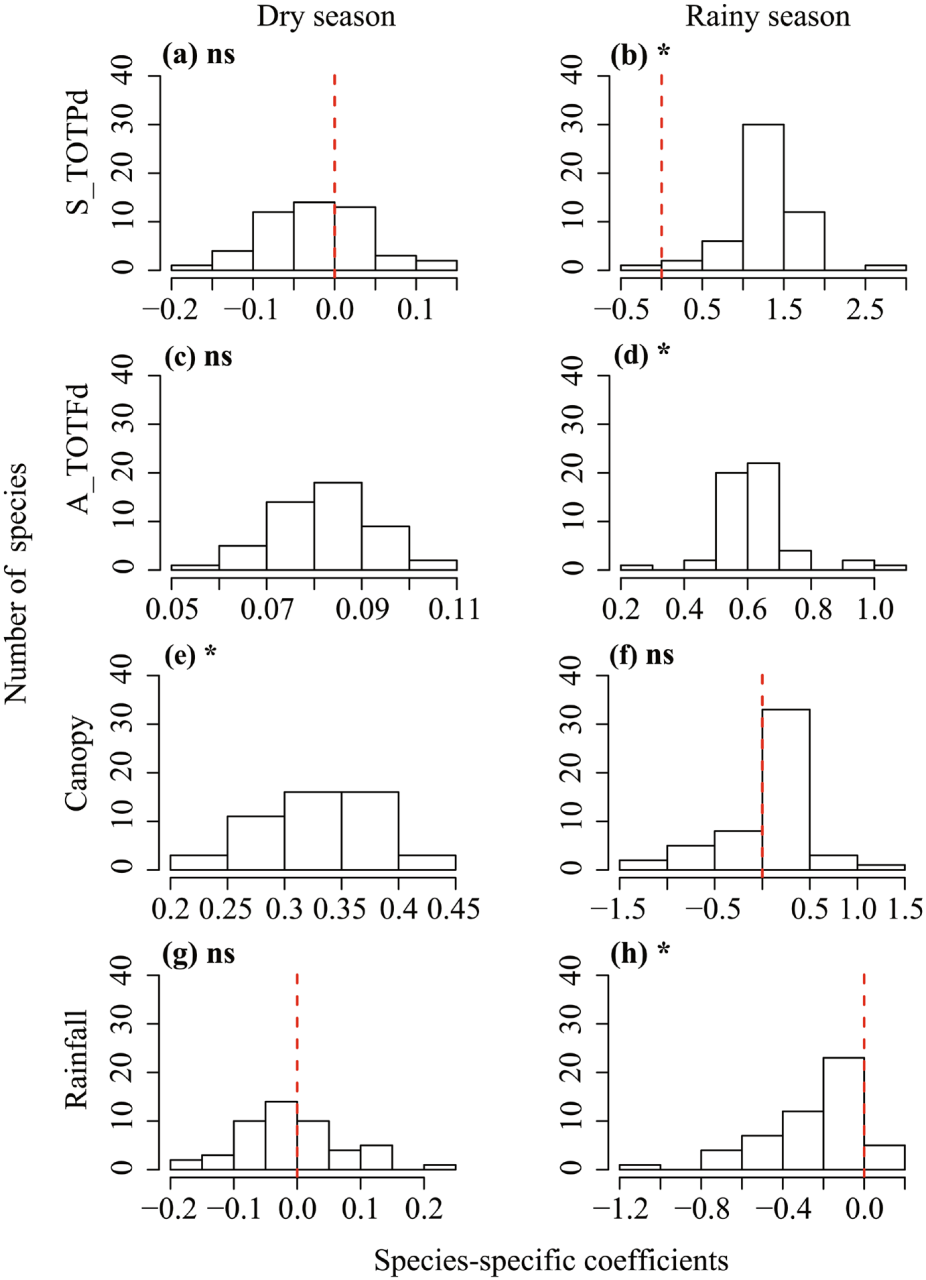
The comparison of the frequency histogram distribution of species‐specific coefficients for total phylogenetic distance of seedling neighbors (S_TOTPd), total functional distance of adult neighbors (A_TOTFd), canopy openness (Canopy), and seasonal rainfall (Rainfall) on seedling survival across dry season (left column) and rainy season (right column). The vertical dashed lines at zero separate negative (left) from positive (right) effects. *p < 0.05; ns, not significant.

We detected significant variation among species in responses to biotic and abiotic factors between dry and rainy seasons (Figure 3, Table S5). The direction of these species‐level effects was consistent with the community‐level patterns reported above. Paired *t*‐tests revealed significant seasonal differences in species‐specific coefficients. The positive effects of S_TOTPd (*t* = −22.70, *p* < 0.001) and A_TOTFd (*t* = −29.54, *p* < 0.001) on survival, as well as the negative effect of seasonal rainfall (*t* = −6.79, *p* < 0.001), were stronger in the rainy season than in the dry season. Conversely, the positive effect of canopy openness (*t* = 6.02, *p* < 0.001) was significantly stronger in the dry season (Figure 3, Table S6).

**FIGURE 4:**
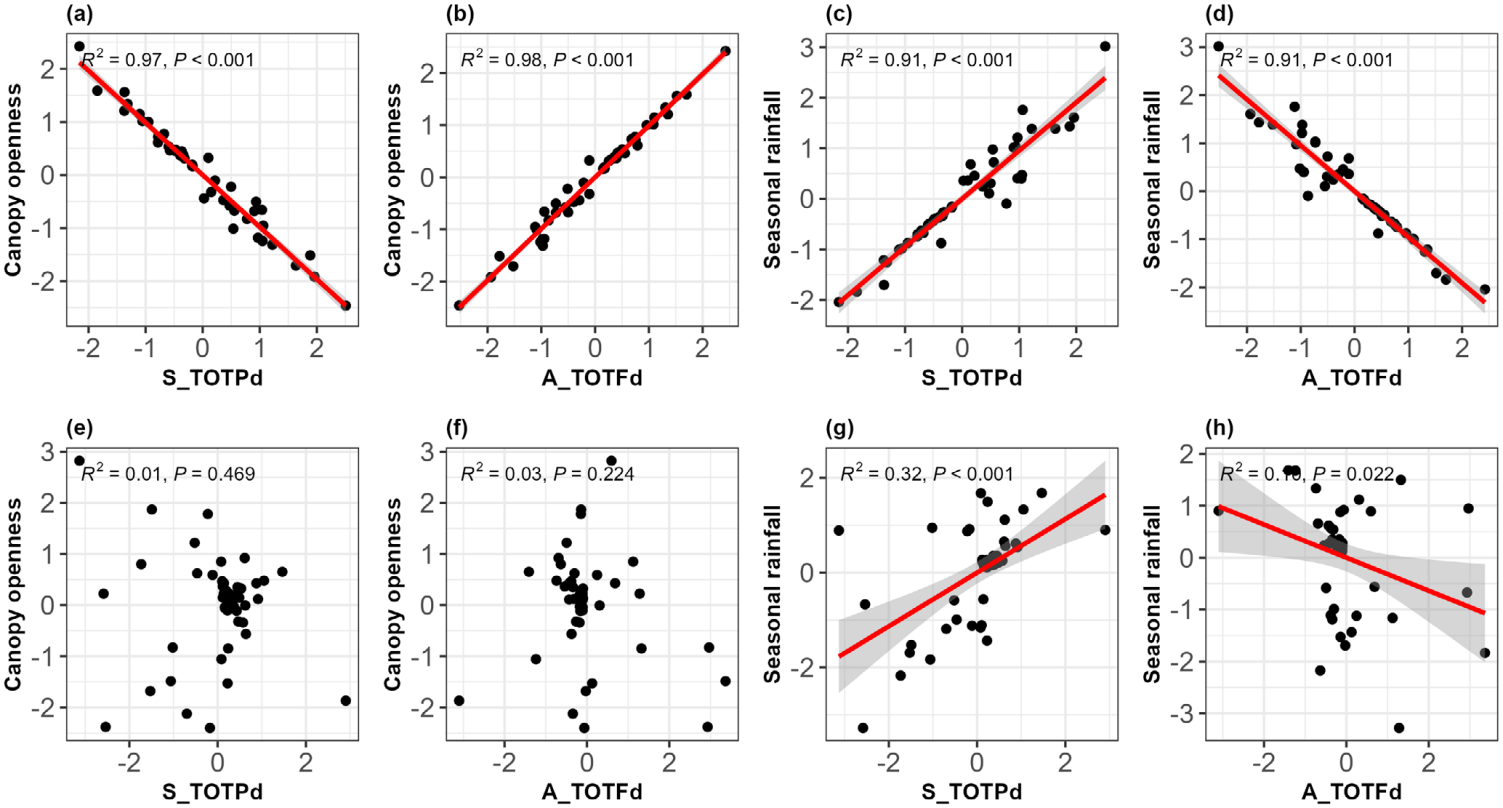
Correlations among species‐specific sensitivities to drivers of seedling survival in dry (a–d) and rainy (e–h) seasons. Gray bands represent 95% confidence intervals. S_TOTPd, total phylogenetic distance of seedling neighbors; A_TOTFd, total functional distance of adult neighbors. Sample size: *N* = 936 seedlings.

Correlation analyses revealed how species' sensitivities to different factors were interrelated (Figure 4). Specifically, a significant negative correlation existed between species‐specific coefficients for canopy openness and S_TOTPd (Figure 4a). The effect of A_TOTFd on survival was moderated by light availability, such that species with higher A_TOTFd coefficients responded more positively to increased canopy openness during the dry season (Figure 4b). In contrast, no significant correlation was detected between canopy openness and either S_TOTPd (Figure 4e) or A_TOTFd (Figure 4f) during the rainy season. Significant positive correlations were found between species‐specific coefficients for rainfall and S_TOTPd in both seasons (Figure 4c,g). However, an opposite, negative interspecific trade‐off was observed between coefficients for rainfall and A_TOTFd (Figure 4d,h). These patterns of correlated sensitivities suggest that a trade‐off in how species respond to different drivers may facilitate their coexistence within the community.

## 4 Discussion

To unravel the dynamics of seedling survival, we adopted an explicit seasonal perspective. By coupling field monitoring with statistical modeling, we quantified how biotic and abiotic drivers interact across dry and rainy seasons in a subtropical forest. Our findings demonstrate that neighbor densities, habitat factors, and climate heterogeneity all play significant roles in seedling survival, but their relative importance shifts markedly between seasons. Concurrently, species‐level survival probabilities showed pronounced seasonal differences. Most importantly, we identified a distinct seasonal trade‐off, wherein the sensitivity of seedling survival to biotic and abiotic drivers reversed between the dry and rainy seasons.

### 4.1 Relative Importance of Variables Shaping Seedling Survival Across Seasons

Biotic and abiotic variables of seedling survival showed clear seasonal shifts, as reflected in corresponding changes in the explanatory power of model predictors. Notably, seedling height had little predictive power for survival in either season, likely because it does not confer a competitive advantage in resource acquisition at this early life stage (Johnson et al. 2017).

CNDD has been widely documented in woody plants across tropical to temperate forests (Chen et al. 2019; Comita et al. 2014; LaManna et al. 2017; Yao et al. 2020; Zhu et al. 2015). Interestingly, there was no detectable CNDD in this plot. This absence does not necessarily imply that conspecific neighbors are unimportant for seedling survival; rather, the CNDD effect may have been masked by habitat filtering or by the generally low densities of conspecific neighbors in our study area (Wu et al. 2016).

Our results reveal strong seasonal variation in the effect of PNDD and FNDD on seedling survival. Their effects were weak in the dry season but became strongly negative in the rainy season, where survival decreased in neighborhoods with phylogenetically or functionally similar neighbors. The observed PNDD pattern was in accordance with previous studies (Metz et al. 2010; Paine et al. 2012; Wang, Wu, Chai, Li, et al. 2024; Webb et al. 2006). Due to niche overlap, closely related individuals would increase resource competition among neighbors (Metz et al. 2010; Pu and Jin 2018; Zhu et al. 2015). In addition, phylogenetically related species tend to share similar traits such as chemical profiles and physical structures, consequently making them susceptible to the same specialized pathogens and natural enemies (Liu et al. 2012; Paine et al. 2012; Queenborough et al. 2009). This “shared susceptibility” facilitates pathogen transmission and strengthens enemy attacks. Similarly, the FNDD effect was in line with the finding that functional dissimilarity among neighbors enhanced seedling survival (Pu et al. 2020). This reinforces the view that niche partitioning, driven by both competitive and enemy‐mediated processes, is a key determinant of seedling survival (Forrister et al. 2019; Kraft et al. 2008). Collectively, our findings illustrate that phylogenetic and functional density dependence, particularly pronounced in the rainy season, may represent general mechanisms that help maintain community composition and diversity (Paine et al. 2012).

Light availability is an indispensable driver of seedling community dynamics, shaping individual survival, distribution, and density (Purves et al. 2007; Zambrano et al. 2019). In our study, the effect of this abiotic factor on seedling survival varied seasonally. Specifically, canopy openness had a strong positive effect on seedling survival in the dry season. This finding is consistent with previous work demonstrating that light availability promotes seedling survival under water‐limited conditions (Yao et al. 2020) and may reflect higher understory light due to reduced cloud cover during the dry season (van Schaik et al. 1993). In contrast, we detected no significant community‐level effect of canopy openness on seedling survival during the rainy season. This lack of a general signal likely arises from divergent species‐specific responses to light; for instance, some species benefit while others are negatively affected by increased openness (Johnson et al. 2017), as revealed by the species‐specific random slopes model (Figure 3h). These patterns illustrate how variation in light‐use strategies among individuals mediates survival in response to seasonal resource changes.

Regarding rainfall, our results contrast with reports from tropical rainforests, where higher dry‐season rainfall is conducive to seedling survival (Song et al. 2024). In our subtropical plot, increased rainy‐season rainfall was associated with lower seedling survival. This discrepancy may reflect differences in forest type. Potential mechanisms for this negative effect include nutrient leaching (Santiago et al. 2004), enhanced pathogen activity (Zheng et al. 2001), or increased herbivore pressure in wetter habitats (Swinfield et al. 2012). As herbivore impacts were not directly quantified in our study, the latter mechanism remains a plausible but untested explanation.

As such, our findings demonstrate that seasonality plays a crucial role in seedling survival. Canopy openness strongly promotes seedling survival in the dry season, whereas seedling survival during the rainy season is more susceptible to phylogenetic relatedness and functional dissimilarity among neighbors, as well as seasonal rainfall.

### 4.2 Species‐Level Variation in Sensitivities to Biotic and Abiotic Drivers Across Seasons

The effects of neighbors, habitat, and seasonal rainfall on seedling survival varied substantially among species and were strongly season‐dependent. In particular, the strength of PNDD and FNDD exhibited greater interspecific variation in the rainy season than in the dry season, implying that phylogenetic relatedness and functional dissimilarity among neighbors may influence the composition of seedlings. The species‐specific seasonal variability of neighborhood effects is critical for understanding differential species survival (Song et al. 2020).

Variation in species' responses to abiotic factors reflects the potential for niche partitioning to maintain species coexistence in forest communities (Johnson et al. 2017; Yao et al. 2020; Zhu et al. 2015). We found significant interspecific differences in responses to canopy openness between seasons, consistent with higher photosynthetically active radiation available during the dry season (Song et al. 2020). Species also varied markedly in their responses to seasonal rainfall, with most showing reduced survival as rainfall increased (Figure 3j). This pattern suggests that only a minority of seedlings perform well under high rainy‐season rainfall, possibly due to generally higher herbivory pressure during this period (Brenes‐Arguedas et al. 2009). Such variation in species‐specific enemy pressure can exert an overall adverse effect at the community level (Johnson et al. 2017).

To further quantify potential trade‐offs, we analyzed species‐level coefficients from the seasonal models. We found evidence for a strong trade‐off. Species whose survival increased more with canopy openness were also more negatively affected by phylogenetic relatedness of heterospecific seedling neighbors and more positively affected by functional dissimilarity of heterospecific adult neighbors. This indicates that light‐demanding seedlings experience stronger neighborhood effects. Furthermore, phylogenetic relatedness appeared to modulate the positive effect of rainfall, whereas a strong negative correlation existed between species‐specific sensitivities to rainfall and functional dissimilarity. Increased precipitation may accelerate soil and nutrient erosion (Song et al. 2024; Yao et al. 2020), resulting in a potential increase in the intensity of competition for soil resources among functionally similar neighbors. As a result, higher functional dissimilarity among species is beneficial for survival. These trade‐offs in survival responses may be one mechanism by which phylogenetic and functional diversity enhance community stability, while also refining our understanding of how tree species respond to concurrent biotic and abiotic pressures. Therefore, environmental context must be explicitly considered when examining survival‐mortality trade‐offs.

### 4.3 Limitations and Future Perspectives

Focusing on leaf functional traits, this study provides key insights into how plant resource‐use strategies shape a major dimension of species coexistence in forest communities. We recognize, however, that survival and competitive strategies are multidimensional (Wang and Huang 2024; Da et al. 2025). Beyond leaf traits, seedling survival is also markedly influenced by belowground traits (e.g., root morphology and architecture; Metz et al. 2023) and reproductive traits (e.g., seed resource allocation and reproductive phenology; Wang et al. 2020). Given the sampling and design constraints of the present work, these belowground and reproductive traits were not measured, which means our interpretation of interspecific resource competition and adaptive strategies may be incomplete. For instance, some species with similar leaf traits may still coexist through differentiation in root architecture or reproductive timing (Wang and Huang 2024). Consequently, integrating the whole‐plant economic spectrum in future studies, especially by examining trade‐offs between both above‐ and below‐ground traits, will provide a more comprehensive perspective on the functional drivers of community assembly.

## 5 Conclusion

Based on seedling dynamic data from a subtropic forest in Southwest China, we found PNDD and FNDD on seedling survival in the rainy season, but not in the dry season. In the dry season, greater canopy openness significantly enhanced seedling survival, whereas in the rainy season, higher rainfall intensity unexpectedly lowered survival. These results collectively indicate a marked seasonal shift in the dominant drivers of seedling dynamics—from strong biotic regulation (PNDD/FNDD) in the rainy season to abiotic facilitation (light availability) in the dry season—thereby demonstrating how temporal niche partitioning may stabilize forest communities.

## Author Contributions

**Liping Wang:** conceptualization (lead), data curation (equal), formal analysis (lead), investigation (equal), methodology (lead), software (lead), validation (lead), visualization (lead), writing – original draft (lead), writing – review and editing (equal). **Junjie Wu:** data curation (equal), funding acquisition (lead), project administration (lead), resources (lead), supervision (lead), writing – review and editing (equal). **Yong Chai:** investigation (equal). **Fengxian Chen:** investigation (equal).

## Conflicts of Interest

The authors declare no conflicts of interest.

## Supporting information


**Figure S1:** Seasonal precipitation patterns.
**Figure S2:** Seedling abundance across seasonal intervals.
**Figure S3:** Diagnostic checks for overdispersion in the seasonal GLMMs.
**Table S1:** Loadings of topography and soil properties on the first three principal components (PCA1, PCA2, and PCA3) in different seasons.
**Table S2:** Akaike's information criterion (AIC) values for density‐dependent models of conspecific adult neighbors at distances of 5, 10, 15, and 20 m from focal seedlings.
**Table S3:** Seedling survival rates of 56 species in total 100 (2 m × 2 m) quadrats over each dry season and rainy season intervals.
**Table S4:** Estimated coefficients (*β*) for all explanatory variables in the dry‐ and rainy‐season GLMMs.
**Table S5:** Species‐level responses from GLMMs.
**Table S6:** Paired *t*‐test results comparing species‐specific coefficients between dry‐ and rainy‐season models.

## Data Availability

The data that support the findings of this study are available from the Dryad Digital Repository: https://doi.org/10.5061/dryad.nk98sf80r (Wang, Wu, Yu, Sun, et al. 2024).
