# Supplementary material for: Seasonal Variation of the Effects of Phylogenetic Relatedness and Functional Similarity Among Heterospecific Neighbors and Habitat on Seedling Survival in a Subtropical Forest in Gaoligong Mountains, Southwest China
Source: Ecol Evol. 2026 Feb 2;16(2):e73021. doi: 10.1002/ece3.73021 (PMC12862240; doi:10.1002/ece3.73021)
Supplement: Supplementary file 1 — Figure S1: Seasonal precipitation patterns. Figure S2: Seedling abundance across seasonal intervals. Figure S3: Diagnostic checks for overdispersion in the seasonal GLMMs. Table S1: Loadings of topography and soil properties on the first three principal components (PCA1, PCA2, and PCA3) in different seasons. Table S2: Akaike's information criterion (AIC) values for density‐dependent models of conspecific adult neighbors at distances of 5, 10, 15, and 20 m from focal seedlings. Table S3: Seedling survival rates of 56 species in total 100 (2 m × 2 m) quadrats over each dry season and rainy season intervals. Table S4: Estimated coefficients (β) for all explanatory variables in the dry‐ and rainy‐season GLMMs. Table S5: Species‐level responses from GLMMs. Table S6: Paired t‐test results comparing species‐specific coefficients between dry‐ and rainy‐season models. [file ECE3-16-e73021-s001.docx]

**Appendix A. Supporting Information**

**Table S1** Loadings of topography and soil properties on the first three principal components (PCA1, PCA2, and PCA3) in different seasons.

| Habitat factor | Dry season | | | Rainy season | | |
| --- | --- | --- | --- | --- | --- | --- |
|  | PCA1 | PCA2 | PCA3 | PCA1 | PCA2 | PCA3 |
| Elevation | 0.809 | 0.203 | 0.225 | 0.809 | 0.206 | 0.228 |
| Convexity | 0.493 | 0.256 | -0.454 | 0.452 | 0.259 | -0.456 |
| Slope | -0.089 | 0.170 | -0.692 | -0.090 | 0.172 | -0.658 |
| Soil pH | 0.001 | 0.001 | 0.001 | 0.001 | 0.001 | 0.001 |
| Soil electrical conductivity (EC) | 0.014 | – | 0.103 | 0.014 | -0.001 | 0.101 |
| Soil organic matter content (C) | 0.292 | -0.922 | -0.207 | 0.294 | -0.920 | -0.217 |
| Soil available phosphorus (AP) | 0.002 | 0.001 | 0.009 | 0.002 | 0.001 | 0.009 |
| Soil available potassium (AK) | 0.005 | 0.002 | 0.023 | 0.005 | 0.002 | 0.022 |
| Soil total nitrogen (TN) | 0.017 | -0.047 | -0.008 | 0.017 | -0.046 | -0.010 |
| Soil total phosphorus (TP) | 0.002 | -0.002 | 0.003 | 0.002 | -0.002 | 0.003 |
| Soil total potassium (TK) | -0.047 | 0.106 | -0.026 | -0.047 | 0.106 | -0.028 |
| Soil temperature | -0.006 | – | -0.015 | 0.015 | -0.008 | 0.037 |
| Soil moisture | 0.083 | -0.001 | 0.457 | 0.082 | -0.025 | 0.498 |
| Variation explained (%) | 32.6 | 20.6 | 13.0 | 33.6 | 20.1 | 13.2 |

Note: – means the term was not included in the PCA axis

**Table S2** Akaike’s information criterion (AIC) values for density‑dependent models of conspecific adult neighbors at distances of 5, 10, 15, and 20 m from focal seedlings.

| Model | Radius distance (m) | AIC |
| --- | --- | --- |
| Height +  conspecific adult neighbor density | 5 | 310.037 |
|  | 10 | 297.747 |
|  | 15 | 295.703 |
|  | 20 | **254.779** |

Note: A bold font indicates the minimum AIC.

**Table S3** Seedling survival rates of 56 species in total 100 (2 m× 2 m) quadrats over each dry season and rainy season intervals. NA denotes that a species was not present over the interval.

| **Species** | **Genus** | **Family** | **Survival rates** | | | |
| --- | --- | --- | --- | --- | --- | --- |
|  |  |  | **First dry season interval** | **First rainy season interval** | **Second dry season interval** | **Second rainy season interval** |
| *Acer pubipetiolatum* | *Acer* | Sapindaceae | 0.88 | 1.00 | 1.00 | 0.86 |
| *Actinodaphne obovata* | *Actinodaphne* | Lauraceae | 0.67 | 1.00 | 1.00 | 0.67 |
| *Ardisia crenata* | *Ardisia* | Primulaceae | 1.00 | 1.00 | 0.93 | 0.85 |
| *Aucuba himalaica* | *Aucuba* | Garryaceae | 1.00 | 1.00 | 1.00 | 1.00 |
| *Beilschmiedia yunnanensis* | *Beilschmiedia* | Lauraceae | 1.00 | 1.00 | 1.00 | 0.83 |
| *Brassaiopsis glomerulata* | *Brassaiopsis* | Araliaceae | 1.00 | 1.00 | 1.00 | 1.00 |
| *Camellia tsaii* | *Camellia* | Theaceae | 0.90 | 1.00 | 1.00 | 0.90 |
| *Camellia taliensis* | *Camellia* | Theaceae | 0.75 | 1.00 | 1.00 | 1.00 |
| *Camellia kissi* | *Camellia* | Theaceae | 1.00 | 1.00 | 1.00 | 1.00 |
| *Castanopsis hystrix* | *Castanopsis* | Fagaceae | 1.00 | 1.00 | 1.00 | 1.00 |
| *Chionanthus ramiflorus* | *Chionanthus* | Oleaceae | 1.00 | NA | NA | NA |
| *Damnacanthus indicus* | *Damnacanthus* | Rubiaceae | 1.00 | 1.00 | 1.00 | 1.00 |
| *Elaeocarpus lacunosus* | *Elaeocarpus* | Elaeocarpaceae | 1.00 | 1.00 | 1.00 | 0.93 |
| *Embelia undulata* | *Embelia* | Primulaceae | 1.00 | 1.00 | 1.00 | 0.90 |
| *Eriobotrya tengyuehensis* | *Eriobotrya* | Rosaceae | 1.00 | 1.00 | 1.00 | 1.00 |
| *Euonymus fortunei* | *Euonymus* | Celastraceae | 1.00 | 1.00 | 0.85 | 0.94 |
| *Euonymus vagans* | *Euonymus* | Celastraceae | 1.00 | 1.00 | 0.67 | 1.00 |
| *Ficus neriifolia* | *Ficus* | Moraceae | 1.00 | 1.00 | 1.00 | 1.00 |
| *Ficus stenophylla* | *Ficus* | Moraceae | 0.50 | 1.00 | 1.00 | 1.00 |
| *Fosbergia shweliensis* | *Fosbergia* | Rubiaceae | 1.00 | 1.00 | 1.00 | 0.95 |
| *Helicia shweliensis* | *Helicia* | Proteaceae | 0.93 | 1.00 | 1.00 | 1.00 |
| *Heptapleurum bodinieri* | *Heptapleurum* | Araliaceae | 1.00 | 1.00 | 0.92 | 1.00 |
| *Hydrangea chinensis* | *Hydrangea* | Hydrangeaceae | 1.00 | 1.00 | 1.00 | 1.00 |
| *Ilex polyneura* | *Ilex* | Aquifoliaceae | 1.00 | 1.00 | 1.00 | 1.00 |
| *Itea omeiensis* | *Itea* | Iteaceae | 1.00 | 1.00 | 0.80 | 1.00 |
| *Iteadaphne caudata* | *Iteadaphne* | Lauraceae | 1.00 | 1.00 | 1.00 | 1.00 |
| *Jasminum grandiflorum* | *Jasminum* | Oleaceae | 1.00 | 0.50 | 1.00 | 1.00 |
| *Lasianthus biermannii* | *Lasianthus* | Rubiaceae | 1.00 | 1.00 | 0.86 | 1.00 |
| *Lindera foveolata* | *Lindera* | Lauraceae | 0.75 | 1.00 | 1.00 | 0.33 |
| *Lithocarpus hancei* | *Lithocarpus* | Fagaceae | 0.94 | 1.00 | 0.89 | 0.94 |
| *Lithocarpus petelotii* | *Lithocarpus* | Fagaceae | 0.80 | 1.00 | 1.00 | 1.00 |
| *Litsea chinpingensis* | *Litsea* | Lauraceae | 1.00 | 1.00 | 1.00 | 0.67 |
| *Litsea elongata* | *Litsea* | Lauraceae | 1.00 | 1.00 | 1.00 | 1.00 |
| *Machilus rufipes* | *Machilus* | Lauraceae | 1.00 | 1.00 | 1.00 | 1.00 |
| *Mahonia oiwakensis* | *Mahonia* | Berberidaceae | 1.00 | 1.00 | 1.00 | 1.00 |
| *Melodinus khasianus* | *Melodinus* | Apocynaceae | 1.00 | 1.00 | 1.00 | 1.00 |
| *Merrilliopanax listeri* | *Merrilliopanax* | Araliaceae | 1.00 | 1.00 | 1.00 | 1.00 |
| *Mycetia yunnanica* | *Mycetia* | Rubiaceae | 1.00 | 1.00 | 1.00 | 0.88 |
| *Myrsine semiserrata* | *Myrsine* | Primulaceae | 1.00 | 1.00 | 0.97 | 1.00 |
| *Neolitsea lunglingensis* | *Neolitsea* | Lauraceae | 1.00 | 0.95 | 0.89 | 0.94 |
| *Neolitsea undulatifolia* | *Neolitsea* | Lauraceae | 1.00 | 1.00 | 1.00 | 0.80 |
| *Neolitsea homilantha* | *Neolitsea* | Lauraceae | 1.00 | 1.00 | 1.00 | 0.67 |
| *Periploca sepium* | *Periploca* | Apocynaceae | 1.00 | 1.00 | 1.00 | 1.00 |
| *Polygala arillata* | *Polygala* | Polygalaceae | 1.00 | 0.89 | 0.88 | 0.43 |
| *Prunus phaeosticta* | *Prunus* | Rosaceae | 1.00 | 1.00 | 1.00 | 1.00 |
| *Psychotria morindoides* | *Psychotria* | Rubiaceae | 1.00 | 1.00 | 0.96 | 0.95 |
| *Rubus chrysobotrys* | *Rubus* | Rosaceae | 1.00 | 1.00 | 1.00 | NA |
| *Sabia parviflora* | *Sabia* | Sabiaceae | 1.00 | 1.00 | 1.00 | 1.00 |
| *Skimmia arborescens* | *Skimmia* | Rutaceae | 1.00 | 1.00 | 1.00 | 1.00 |
| *Symplocos stellaris* var. *aenea* | *Symplocos* | Symplocaceae | 1.00 | 1.00 | 0.97 | 1.00 |
| *Symplocos ramosissima* | *Symplocos* | Symplocaceae | 1.00 | 1.00 | 0.95 | 0.95 |
| *Symplocos theophrastifolia* | *Symplocos* | Symplocaceae | 1.00 | 1.00 | 1.00 | 0.92 |
| *Symplocos glauca* | *Symplocos* | Symplocaceae | 1.00 | 1.00 | 1.00 | 1.00 |
| *Trachelospermum bodinieri* | *Trachelospermum* | Apocynaceae | 1.00 | 1.00 | 1.00 | 1.00 |
| *Trevesia palmata* | *Trevesia* | Araliaceae | 0.80 | 0.50 | 1.00 | 0.75 |

**Table S4** Estimated coefficients (β) for all explanatory variables in the dry‑ and rainy‑season GLMMs. Variables included conspecific seedling (S_con) and adult (A_con) neighbor densities, phylogenetic distances of heterospecific seedling (S_TOTPd) and adult (A_TOTPd) neighbors, functional distances of heterospecific seedling (S_TOTFd) and adult (A_TOTFd) neighbors, canopy openness (Canopy), the first three principal components of habitat (PCA1–PCA3), and seasonal rainfall (Rainfall).

| Explanatory variables | Dry season | | | Rainy season | | |
| --- | --- | --- | --- | --- | --- | --- |
|  | *β* | *CI* | *P* | *β* | *EC* | *P* |
| Height | 0.228 | [–0.013, 0.469] | 0.064 | 0.185 | [–0.146, 0.516 | 0.273 |
| S_con | 0.155 | [–0.151, 0.461] | 0.321 | 0.047 | [–0.365, 0.459] | 0.824 |
| A_con | 0.373 | [–0.106, 0.853] | 0.127 | 0.097 | [–0.333, 0.527] | 0.658 |
| S_TOTPd | –0.005 | [–0.307, 0.296] | 0.972 | 0.939 | [0.173, 1.706] | **0.016** |
| A_TOTPd | –0.146 | [–0.608, 0.315] | 0.534 | –0.571 | [–1.143, 0.001] | 0.051 |
| S_TOTFd | 0.032 | [–0.342, 0.405] | 0.868 | –0.330 | [–0.808, 0.147] | 0.175 |
| A_TOTFd | 0.060 | [–0.329, 0.450] | 0.761 | 0.681 | [0.229, 1.133] | **0.003** |
| Canopy | 0.315 | [0.037, 0.592] | **0.026** | 0.135 | [–0.205, 0.474] | 0.436 |
| PCA1 | 0.021 | [–0.259, 0.302] | 0.881 | 0.020 | [–0.313, 0.352] | 0.908 |
| PCA2 | –0.243 | [–0.544, 0.058] | 0.113 | 0.185 | [–0.175, 0.546] | 0.314 |
| PCA3 | 0.064 | [–0.163 ,0.291] | 0.582 | 0.021 | [–0.256, 0.298] | 0.880 |
| Rainfall | 0.046 | [–0.185 ,0.278] | 0.695 | –0.412 | [–0.748, –0.077] | **0.016** |

Note: Bold values are statistically significant (*P* < 0.05), as determined by 95% confidence intervals (CI) that do not include zero.

**Table S5** Species‑level responses from GLMMs. Results show the estimated effects on seedling survival of phylogenetic distance of heterospecific seedling neighbors (S_TOTPd), functional distance of heterospecific adult neighbors (A_TOTFd), canopy openness, and seasonal rainfall.

| Seasons | Variables | Slope | SE | *Z* value | *P*-value | *P*_adj_-value |
| --- | --- | --- | --- | --- | --- | --- |
| Dry season | S_TOTPd | -0.029 | 0.164 | -0.176 | 0.860 | 0.932 |
|  | A_TOTFd | 0.083 | 0.227 | 0.366 | 0.714 | 0.939 |
|  | Canopy openness | 0.335 | 0.175 | 1.919 | **0.035** | **0.047** |
|  | Rainfall | -0.019 | 0.176 | -0.108 | 0.914 | 0.936 |
| Rainy season | S_TOTPd | 1.370 | 0.565 | 2.427 | **0.025** | **0.049** |
|  | A_TOTFd | 0.605 | 0.349 | 1.733 | **0.009** | **0.022** |
|  | Canopy openness | 0.044 | 0.294 | 0.150 | 0.881 | 0.954 |
|  | Rainfall | -0.228 | 0.264 | -0.863 | **0.031** | **0.048** |

Note: The table provides, for each predictor, the slope estimate (Slope), standard error (SE), test statistic (Z value), raw *P*-value, and false discovery rate (FDR)‑adjusted *P*_adj_‑value. Estimates in bold are statistically significant (*P* < 0.05).

**Table S6** Paired *t*-test results comparing species-specific coefficients between dry‑ and rainy‑season models. Tests were performed for coefficients of phylogenetic distance of heterospecific seedling neighbors (S_TOTPd), functional distance of heterospecific adult neighbors (A_TOTFd), canopy openness, and seasonal rainfall.

| Variables | *t* | *P*-value |
| --- | --- | --- |
| S_TOTPd | -22.70 | <0.001 |
| A_TOTFd | -29.54 | <0.001 |
| Canopy openness | 6.02 | <0.001 |
| Rainfall | -6.79 | <0.001 |


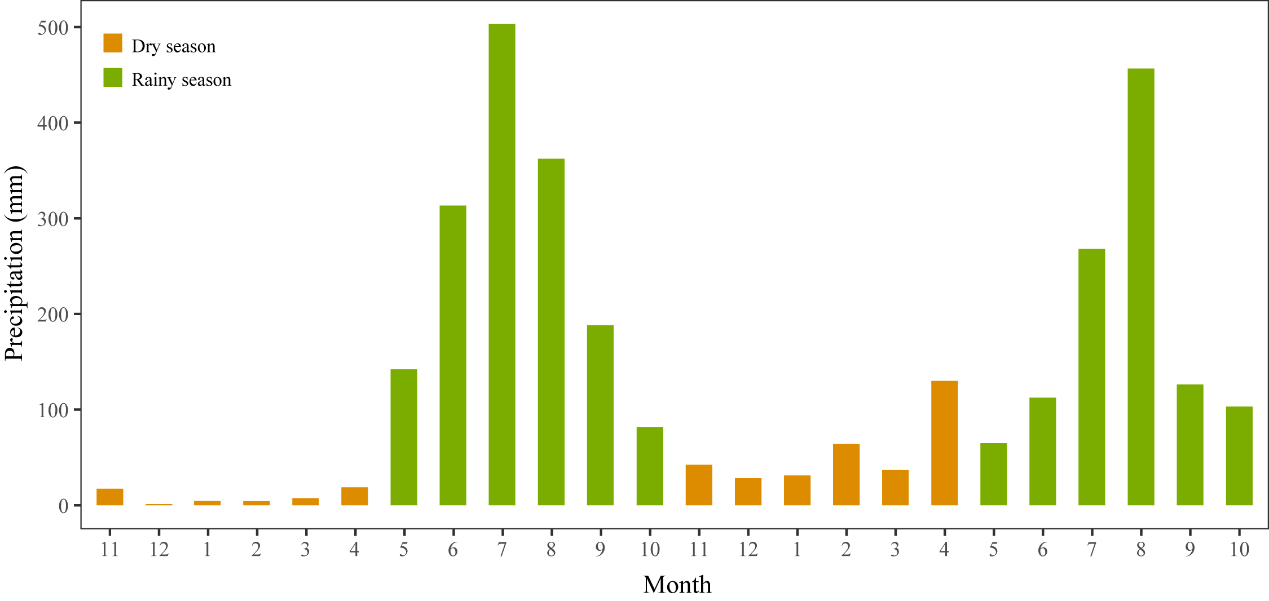


**Figure S1** Seasonal precipitation patterns. Monthly precipitation during two complete dry‑ and rainy‑season cycles (November 2020–October 2022) in the Gaoligong Mountains.


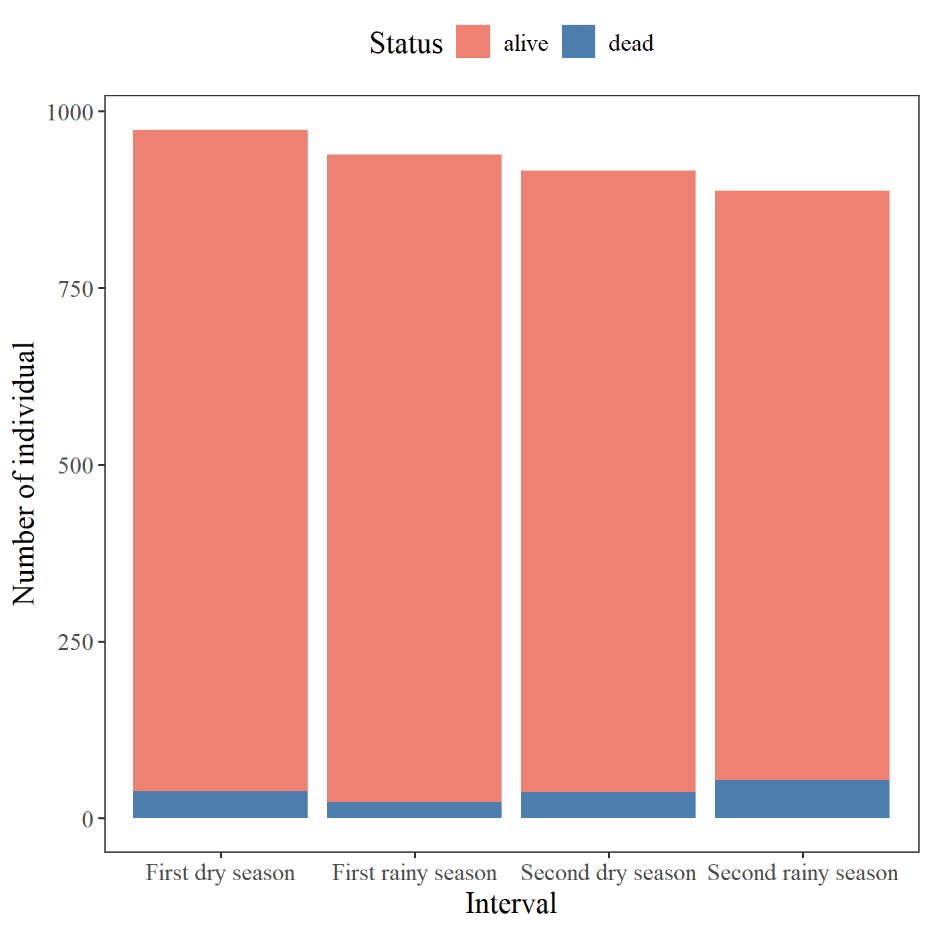


**Figure S2** Seedling abundance across seasonal intervals. Number of seedlings recorded during successive dry‑ and rainy‑season census periods.


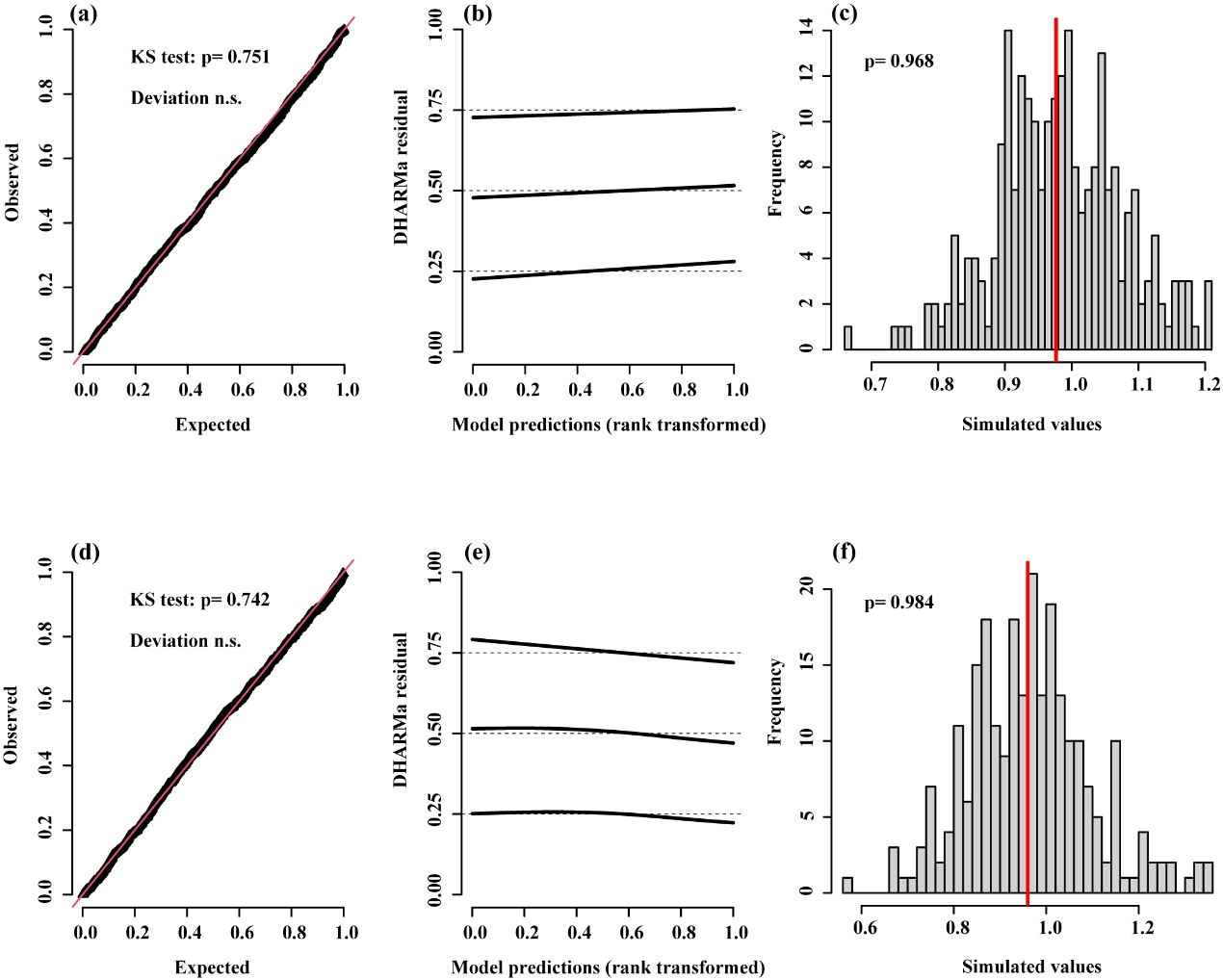


**Figure S3** Assessment of overdispersion in seasonal GLMMs using DHARMa. Diagnostic plots from nonparametric dispersion tests for the dry‑season (a‑c) and rainy‑season (d‑f) models. (a), (b), (d), (e) show quantile‑quantile (QQ) plots comparing observed residuals against simulated residuals under the fitted model; the red line indicates the theoretical expectation under the null hypothesis. (c), (f) display the distribution of simulated dispersion statistics (histogram); the vertical red line marks the observed dispersion value. The reported p-value tests the null hypothesis of no overdispersion; p > 0.05 indicates that the model assumption is met.
